# Severe Pediatric Adenovirus 7 Disease in Singapore Linked to Recent Outbreaks across Asia

**DOI:** 10.3201/eid2107.141443

**Published:** 2015-07

**Authors:** Oon Tek Ng, Koh Cheng Thoon, Hui Ying Chua, Natalie Woon Hui Tan, Chia Yin Chong, Nancy Wen Sim Tee, Raymond Tzer Pin Lin, Lin Cui, Indumathi Venkatachalam, Paul Anantharajah Tambyah, Jonathan Chew, Raymond Kok Choon Fong, Helen May Lin Oh, Prabha Unny Krishnan, Vernon Jian Ming Lee, Boon Huan Tan, Sock Hoon Ng, Pei Jun Ting, Sebastian Maurer-Stroh, Vithiagaran Gunalan, Wei Xin Khong

**Keywords:** Human adenovirus, viruses, Ad7, outbreak, Asia, China, Singapore, Malaysia, invasive disease, death, mechanical ventilation

## Abstract

During November 2012–July 2013, a marked increase of adenovirus type 7 (Ad7) infections associated with severe disease was documented among pediatric patients in Singapore. Phylogenetic analysis revealed close genetic links with severe Ad7 outbreaks in China, Taiwan, and other parts of Asia.

The number of pediatric inpatients increased from 32 during January–July 2012 to 200 cases during January–July 2013 (Figure 1). 

**Figure 1 F1:**
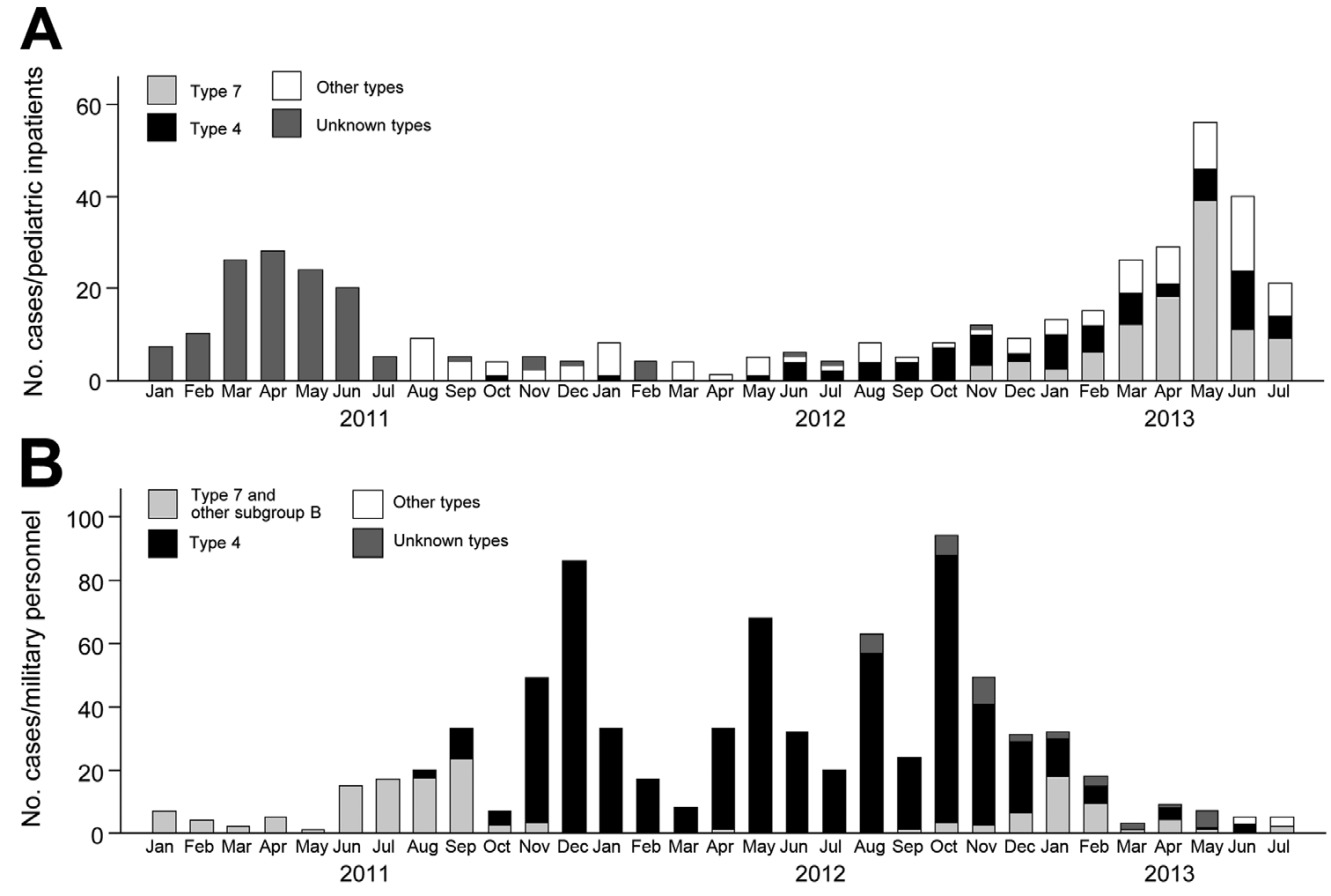
Number of adenovirus cases by month, January 2011–July 2013, Singapore. A) The first confirmed case of adenovirus type 7 was reported in November 2012 in KK Women’s and Children’s Hospital. The number of human adenovirus cases among the pediatric inpatient population increased and peaked in May 2013. B) A retrospective examination of the military surveillance data revealed the first appearance of adenovirus type 7 among military personnel in September 2012 and a small increase and decline over the next few months.

(Table 1). A total of 54 patients had invasive infections and 134 had noninvasive infections (Table 2). 

**Table 1 T1:** Demographics and clinical features of 188 hospitalized children with adenovirus, by age group, Singapore*

Characteristics	Values


**Table 2 T2:** Number and percentages of 188 hospitalized pediatric adenovirus case-patients with noninvasive and invasive infection, by key characteristics, and risk factors associated with invasive infection, Singapore*

Characteristics	Noninvasive infection, n = 134	Invasive infection, n = 54	Univariate analysis			Multivariate analysis†	
			Crude OR (95% CI)	p value		Adjusted OR (95% CI)	p value


(GenBank accession nos. KP729815–KP729824) (Figure 2) 

**Figure 2 F2:**
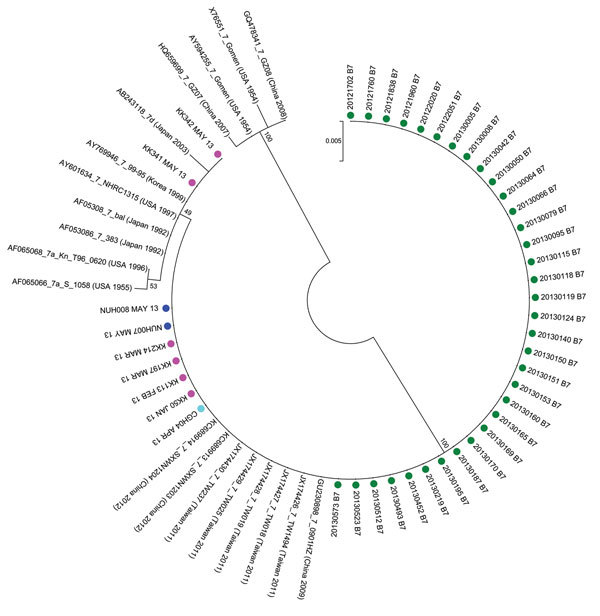
Phylogenetic analysis of adenovirus type 7 (Ad7) sequences from this study based on sequenced Ad7 hexon gene hypervariable regions 1–6 (Ad7 reference Gomen AY594255 hexon gene nt 324 to 1123). The phylogenetic relationships between Ad7 isolates in this study were inferred by using the maximum-likelihood method based on the Tamura-Nei model (*8*). Initial trees for the heuristic search were obtained by applying the neighbor-joining method to a matrix of pairwise distances estimated by using the maximum-composite likelihood approach. Tree is drawn to scale; branch lengths are measured in the number of substitutions per site, the rate of which was assumed to be uniformly distributed. The analysis involved 63 nt sequences. Green indicates isolates from military personnel, dark blue indicates isolates from patients in National University Hospital, pink indicates isolates from patients in KK Women’s and Children’s Hospital, and light blue indicates isolates from patients in Changi General Hospital. The sequence labeled as “AY594255_7_Gomen (USA 1954)” represents 1 of the published Gomen sequences (AY594255). “_7” is added to GenBank accession numbers to denote that these are Ad7 sequences. All other strains shown are published Ad7 reference isolates. Phylogenetic analyses were conducted by using MEGA6 (*9*). KK341 and KK342 had a single thymine-to-cytosine mutation (Ad7 reference Gomen AY594255 hexon gene nt 780). The remaining 41 isolates from Singapore had 100% nucleotide identity.

## References
